# Specific Micronutrient Intake Association with Diabetic Neuropathy Severity in Adults with Type 2 Diabetes

**DOI:** 10.3390/nu18132134

**Published:** 2026-07-01

**Authors:** Claudiu Cobuz, Mădălina Ungureanu-Iuga, Alina Cornea, Iuliana Costescu, Maricela Cobuz

**Affiliations:** 1 Faculty of Medicine and Biological Sciences, Ştefan cel Mare University of Suceava, 13th Universităţii Street, 720229 Suceava, Romania; 2 Integrated Center for Research, Development and Innovation in Advanced Materials, Nanotechnologies, and Distributed Systems for Fabrication and Control (MANSiD), Ştefan cel Mare University of Suceava, 13th Universităţii Street, 720229 Suceava, Romania; 3 “Sfântul Ioan cel Nou” Emergency Clinical Hospital, 720224 Suceava, Romania

**Keywords:** micronutrients, food frequency questionnaire, glycemic control, food consumption, severity of diabetic neuropathy

## Abstract

Background/Objectives: Diabetic neuropathy is a prevalent complication of type 2 diabetes mellitus (T2DM), but the contribution of dietary factors to neuropathy severity remains insufficiently characterized. This study investigated associations between dietary patterns, nutrient intake, and neuropathy severity in 300 adults with T2DM from Northeastern Romania. Methods: Dietary intake was assessed using a validated food frequency questionnaire, and five dietary patterns were derived using principal component analysis. Neuropathy severity was analyzed as an ordinal outcome using logistic regression models adjusted for age, sex, HbA1c, diabetes duration, and treatment. Results: Higher adherence to Western/fast-food and alcohol and animal fat dietary patterns was associated with greater neuropathy severity in unadjusted analyses, whereas a healthy/prudent pattern showed inverse associations; however, these relationships were attenuated after multivariable adjustment. In contrast, higher intakes of protein (OR = 0.98, 95% CI: 0.97–0.99), magnesium (OR = 0.99, 95% CI: 0.98–1.00), zinc (OR = 0.80, 95% CI: 0.69–0.94), vitamin B3 (OR = 0.94, 95% CI: 0.89–0.99), and vitamin B12 (OR = 0.76, 95% CI: 0.62–0.93) remained independently associated with lower neuropathy severity after adjustment. Conclusions: These findings suggest that specific nutrient intakes may be more strongly associated with diabetic neuropathy severity than overall dietary patterns, highlighting potential nutritional targets for neuropathy risk reduction and clinical management in patients with T2DM.

## 1. Introduction

Diabetic neuropathy is one of the most common and disabling chronic complications of type 2 diabetes mellitus, contributing substantially to pain, sensory loss, gait impairment, foot ulceration, and reduced quality of life. Distal symmetric polyneuropathy remains highly prevalent and is closely linked to long-term glycemic exposure and disease duration [1,2,3]. Current standards of care continue to emphasize chronic hyperglycemia as the major modifiable driver of microvascular complications, including neuropathy [1].

The pathogenesis of diabetic neuropathy is complex and multifactorial. In addition to glucotoxicity, several interrelated mechanisms have been implicated, including oxidative stress, mitochondrial dysfunction, chronic low-grade inflammation, endothelial impairment, and microvascular injury [2,3,4]. These pathways may act synergistically to promote neuronal damage and impaired nerve repair, suggesting that diabetic neuropathy evolves within a broader metabolic and inflammatory milieu rather than as a consequence of hyperglycemia alone [2,3,4].

Within this context, nutrition has attracted increasing attention as a potentially relevant modifier of neuropathy risk and severity. Traditionally, nutritional research in diabetes has focused on isolated nutrients or on general dietary recommendations. However, dietary pattern analysis may provide a more comprehensive representation of habitual intake by capturing the combined effects of foods consumed together in daily life. Recent evidence suggests that healthier dietary patterns, such as Mediterranean, plant-based, and low-glycemic-index approaches, are associated with improved glycemic and cardiometabolic outcomes in people with type 2 diabetes, whereas Western-type patterns rich in refined carbohydrates, processed foods, and saturated fats are generally associated with a less favorable metabolic profile [5,6]. Greater adherence to the Mediterranean dietary pattern has been associated with a reduction in type 2 diabetes incidence of up to 23%, likely through improvements in insulin sensitivity, reduced systemic inflammation, better weight management, and favorable modulation of the gut microbiota [7,8]. The protective effect of the Mediterranean diet against type 2 diabetes is likely mediated through several complementary mechanisms, including reduced oxidative stress and chronic inflammation, improved insulin sensitivity through adequate magnesium intake, and enhanced glycemic control via the high fiber content of whole grains, legumes, fruits, and vegetables, which slows gastric emptying and glucose absorption [7]. Furthermore, the diet supports healthy weight management and abdominal fat reduction, while bioactive compounds such as polyphenols may further improve insulin signaling and overall metabolic function [7]. In contrast, Western dietary patterns characterized by high intakes of refined carbohydrates, processed foods, red and processed meats, and saturated fats have been consistently associated with increased insulin resistance, chronic low-grade inflammation, and a higher risk of type 2 diabetes development and progression [9]. At the same time, specific nutrients may also be biologically relevant to peripheral nerve health. Experimental and clinical evidence suggests that magnesium, zinc, and neurotropic B vitamins may influence glucose metabolism, oxidative balance, inflammatory signaling, myelin integrity, and nerve regeneration [4,10]. Magnesium is involved in neuronal excitability and energy metabolism, while zinc contributes to antioxidant defense and modulation of neuroinflammatory pathways [11,12]. Neurotropic B vitamins, particularly vitamins B1, B6, and B12, play essential roles in myelin synthesis and maintenance, neurotransmitter production, and axon repair, all of which are critical for normal peripheral nerve function and regeneration [13]. Among these, vitamin B12 deserves particular attention in type 2 diabetes because long-term metformin therapy has been consistently associated with reduced vitamin B12 levels, and vitamin B12 deficiency may contribute to neuropathic symptoms or aggravate pre-existing diabetic neuropathy [14,15].

Although the relationship between diet and glycemic control in type 2 diabetes has been widely studied, the relative contribution of overall dietary patterns versus individual nutrient intake to diabetic neuropathy severity remains insufficiently defined [3,5,6]. In particular, it is still unclear whether dietary patterns primarily reflect the broader metabolic burden of disease, while certain nutrients retain more specific associations with neuropathic severity. In this context, the aim of the present study was to evaluate the association between dietary patterns, nutrient intake, and diabetic neuropathy severity in patients with type 2 diabetes. This study provides novel insights into the relationship between diet and diabetic neuropathy by integrating both data-driven dietary patterns and detailed nutrient-level analyses in relation to neuropathy severity, modeled as an ordinal outcome. Unlike most previous studies that have focused either on predefined dietary indices or on single nutrients, without considering neuropathy severity, our approach captures real-world eating behaviors while simultaneously identifying specific nutritional components associated with disease severity. Additionally, the study contributes evidence from an underrepresented Eastern European population, providing context-specific dietary data that are rarely captured in the existing literature. While the potential roles of individual micronutrients such as vitamin B12, magnesium, and zinc in diabetic neuropathy have been previously reported, few studies have simultaneously examined these nutrient-level associations alongside derived dietary patterns and neuropathy severity within the same population. Together, these elements position the study as a comprehensive and methodologically robust contribution to nutritional epidemiology in diabetic complications.

## 2. Materials and Methods

### 2.1. Data Collection

We conducted a cross-sectional observational study including 300 adults diagnosed with type 2 diabetes mellitus and sensory–motor diabetic peripheral polyneuropathy. Participants were evaluated at the Department of Diabetes, Nutrition, and Metabolic Diseases, as well as the affiliated outpatient clinic, of the “Sfântul Ioan cel Nou” County Emergency Clinical Hospital in Suceava. The study was conducted in accordance with the Declaration of Helsinki and approved by the Ethics Committee of the “Sfântul Ioan cel Nou” Emergency Clinical Hospital, Suceava, Romania (Approval No. 24/27 April 2026) for studies involving humans.

Inclusion criteria were age ≥18 years, diagnosis of type 2 diabetes mellitus, and the presence of sensory–motor diabetic peripheral polyneuropathy confirmed using the Toronto Clinical Scoring System (TCSS), as documented in the medical records. The TCSS is a validated clinical instrument used to assess both the presence and severity of diabetic polyneuropathy. Patients with incomplete demographic, clinical, or dietary data were excluded, as were those with incomplete or unreliable Food Frequency Questionnaires (FFQ) that did not allow accurate estimation of nutrient intake. Additionally, participants with implausible total energy intake (<800 kcal/day or >4200 kcal/day) were excluded, in accordance with commonly applied criteria in FFQ-based studies [16].

Demographic and clinical data were collected from medical records and study documentation and included age, sex, area of residence, education level, duration of diabetes, most recent HbA1c value, antidiabetic treatment, and TCSS score. The neuropathy level was established as previously described in the literature [17].

### 2.2. Dietary Intake Assessment

Habitual dietary intake over the previous four weeks was assessed using a semi-quantitative Food Frequency Questionnaire (Figure S1, Supplementary Material), validated for the adult Romanian population [18]. The FFQ was administered by trained personnel through standardized interviews to minimize reporting bias. The instrument includes 53 food items and was previously validated against 7-day dietary records, demonstrating adequate accuracy in estimating energy and nutrient intake. For each food item, participants reported the usual frequency of consumption (daily, weekly, or monthly) and the quantity consumed per occasion, using predefined standard portion sizes or common household measures (e.g., slice, cup, tablespoon).

### 2.3. Conversion of FFQ Data into Quantitative Intake

FFQ data were converted into daily quantitative intake using established methodologies applied in nutritional epidemiology [16]. Consumption frequencies were transformed into daily equivalents (times/day), and reported quantities were expressed as portion-size multipliers relative to standard servings for each food item. The estimated daily intake for each food item (g/day) was calculated as follows (Equation (1)):

(1) $$Daily\;intake\;\left(\frac{g}{day}\right)=frequency\;\left(\frac{times}{day}\right)\times portion\;multiplier\;\times \;standard\;portion\;size\;(g)$$

### 2.4. Estimation of Macro- and Micronutrient Intake

Nutritional composition values were assigned to each food item using the USDA Food and Nutrient Database for Dietary Studies, expressed per 100 g or 100 mL. Daily nutrient intake was calculated as follows (Equation (2)):

(2) $$Nutrient\;intake=\;\frac{daily\;intake\;\times nutrient\;content\;per\;100\;g\;or\;100\;mL}{100}$$

Based on these calculations, total daily energy intake (kcal/day), macronutrients (protein, total fat, saturated fatty acids, carbohydrates, total sugars, and fiber), and micronutrients (calcium, magnesium, iron, zinc, sodium, potassium, vitamins A, C, D, E, B1, B2, B3, B6, folate, and vitamin B12) were estimated for each participant.

Total daily energy and nutrient intake were obtained by summing contributions from all consumed food items. Dietary variables were subsequently aggregated at the participant level in the final analytical dataset.

### 2.5. Energy Adjustment

To reduce the influence of total energy intake on dietary comparisons, nutrients were analyzed both as absolute daily values and as energy-adjusted values per 1000 kcal, calculated using the following formula (Equation (3)):

(3) $$Energy-adjusted\;intake=\;\frac{total\;nutrient\;intake}{total\;energy\;intake}\times 1000$$

This approach allows assessment of nutrient density independently of total caloric intake and is widely used in FFQ-based nutritional epidemiology studies.

### 2.6. Statistics

Principal component analysis (PCA) with Varimax rotation was applied to identify dietary food patterns. The sampling adequacy was acceptable (KMO = 0.552), and Bartlett’s test of sphericity was statistically significant (χ^2^ = 19,536.961, *p* < 0.001), indicating that the correlation matrix was suitable for factor analysis. The number of components retained was based on the scree plot, cumulative explained variance, and interpretability of the factors. Cronbach’s alpha (Table S1, Supplementary Material) indicated acceptable internal reliability between the items of each factor (α > 0.7). A five-component solution was selected, explaining 22% of the total variance. The first pattern (Western/fast-food pattern) was characterized by high consumption of processed meats (sausages, cold cuts), fast food, pizza, chips, fried potatoes, sugary beverages, and alcoholic beer, reflecting an energy-dense, highly processed dietary behavior. The second pattern (healthy/prudent pattern) included high intake of vegetables (raw and cooked), legumes, fresh fruits, fish (cooked and canned), and low-fat dairy products, along with lower consumption of certain processed potato products, representing a nutrient-rich dietary pattern. The third pattern (refined grains and starches pattern) was defined by high consumption of white bread, pasta, white rice, and boiled potatoes and low consumption of whole grains such as dark bread and brown rice. The fourth pattern (light meal and sweet spreads pattern) was characterized by higher consumption of tea, semi-skimmed milk, toast, and sweet spreads such as jam and biscuits, combined with lower consumption of meat, poultry, and fried potato products, reflecting a lighter, snack-oriented dietary behavior. The fifth pattern (alcohol and animal fat pattern) was characterized by high consumption of wine and spirits, along with full-fat dairy products (full-fat milk and cheese) and red and processed meats, indicating a dietary pattern combining alcohol intake with energy-dense, animal-derived foods.

Descriptive statistics were determined to assess the characteristics of the cohort. χ^2^ test was used for comparisons of the categorical variables, while the Mann–Whitney test was used for the numerical ones (*p* < 0.05).

Principal component analysis (PCA) with Varimax rotation was used for factor reduction. The number of components retained was based on the scree plot, cumulative explained variance, and interpretability of the factors. Cronbach’s alpha was used to test internal reliability between pattern items. Dietary pattern scores were calculated by averaging the standardized intake variables that loaded on each component identified by principal component analysis. The resulting scores were standardized (z-scores) to allow comparability across patterns. Differences in dietary pattern scores and nutrients across neuropathy severity groups (neuropathy levels 1–3) were assessed using the Jonckheere–Terpstra test.

Ordinal logistic regression analyses were performed to assess the association between each dietary pattern or nutrient and neuropathy severity. Separate models were first fitted for each dietary pattern or nutrient, followed by multivariable models adjusted for diabetes duration, HbA1c, age, sex, and treatment. Data processing was carried out using SPSS software (IBM Statistics, trial version).

## 3. Results

### 3.1. Characteristics of the Population

Neuropathy severity was significantly associated with longer diabetes duration and higher HbA1c levels (*p* < 0.001 for both), indicating that disease progression and poor glycemic control contribute to more advanced neuropathy (Table 1). Treatment patterns also differed significantly, with increased use of insulin and combination therapy in higher neuropathy stages (*p* < 0.001). In contrast, age and sex were not significantly associated with neuropathy severity (*p* = 0.28 and *p* = 0.70, respectively).

### 3.2. Food Patterns

**Figure 1:**
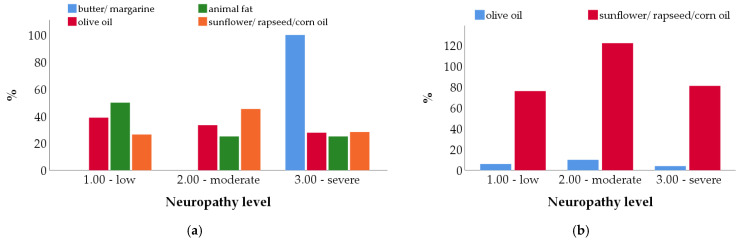
Distribution of cooking fat types according to neuropathy severity: (**a**) Type of fat used for meat cooking. (**b**) Type of fat used for vegetable cooking. Values are expressed as percentages of participants within each neuropathy category.

The Jonckheere–Terpstra test (Table 2) revealed a significant increasing trend across neuropathy severity for the Western/fast-food dietary pattern (Z = 2.24, *p* = 0.03) and refined grains and starches pattern (Z = 1.99, *p* = 0.04) and a borderline significant trend for the alcohol and animal fat pattern (Z = 1.92, *p* = 0.05). In contrast, the healthy/prudent dietary pattern showed a significant decreasing trend with increasing neuropathy severity (Z = −3.06, *p* = 0.01). No significant trend was observed for the light meal and sweet spreads pattern (*p* = 0.76). Figure 1 illustrates the types of fats used for cooking according to neuropathy severity. The distribution of fats used for meat preparation is presented in Figure 1a, whereas Figure 1b shows the fats used for vegetable cooking.

Patients with severe neuropathy showed a higher proportion of butter/margarine consumption, while animal fat intake appeared lower compared to those with mild neuropathy (Figure 1). Vegetable oil consumption demonstrated variable patterns, with a tendency toward higher intake in the moderate neuropathy group. However, these differences were not statistically significant based on Jonckheere–Terpstra tests (*p* > 0.05).

All models were statistically significant at the global level (*p* < 0.05). In unadjusted ordinal logistic regression models (Table 3), several dietary patterns were associated with neuropathy severity, including the Western/fast-food pattern (OR = 1.26, *p* = 0.03), the healthy/prudent pattern (OR = 0.70, *p* = 0.01), and the alcohol and animal fat pattern (OR = 1.26, *p* = 0.03), while the refined carbohydrate pattern showed a borderline association (OR = 1.24, *p* = 0.05). However, after full adjustment for age, sex, treatment, diabetes duration, and HbA1c, none of these associations remained statistically significant. For example, the Western pattern (OR = 0.97, *p* = 0.85), healthy/prudent pattern (OR = 0.88, *p* = 0.42), refined carbohydrate pattern (OR = 1.04, *p* = 0.80), and alcohol and animal fat pattern (OR = 1.23, *p* = 0.22) were all no longer associated with neuropathy severity.

### 3.3. Nutrient Intake

The Jonckheere–Terpstra test revealed significant trends across neuropathy levels for several nutrients and clinical variables (Table 4). A significant decreasing trend (negative Z) was observed for protein (*p* = 0.001), magnesium (*p* = 0.003), zinc (*p* = 0.012), vitamin B3 (*p* = 0.014), and vitamin B12 (*p* = 0.005), indicating that intake levels of these nutrients decrease with increasing neuropathy severity.

In contrast, HbA1c (*p* < 0.001), diabetes duration (*p* < 0.001), and treatment (*p* < 0.001) showed strong increasing trends (positive Z), suggesting that poorer glycemic control, longer disease duration, and treatment intensity are associated with more severe neuropathy. No significant trends were observed for lipids, fiber, calcium, iron, sodium, potassium, most vitamins (A, C, D, E, B1, and folate), age, sex, or carbohydrate intake, although borderline trends were noted for vitamin B2 (*p* = 0.076), vitamin B6 (*p* = 0.067), and carbohydrates (*p* = 0.056).

**Figure 2:**
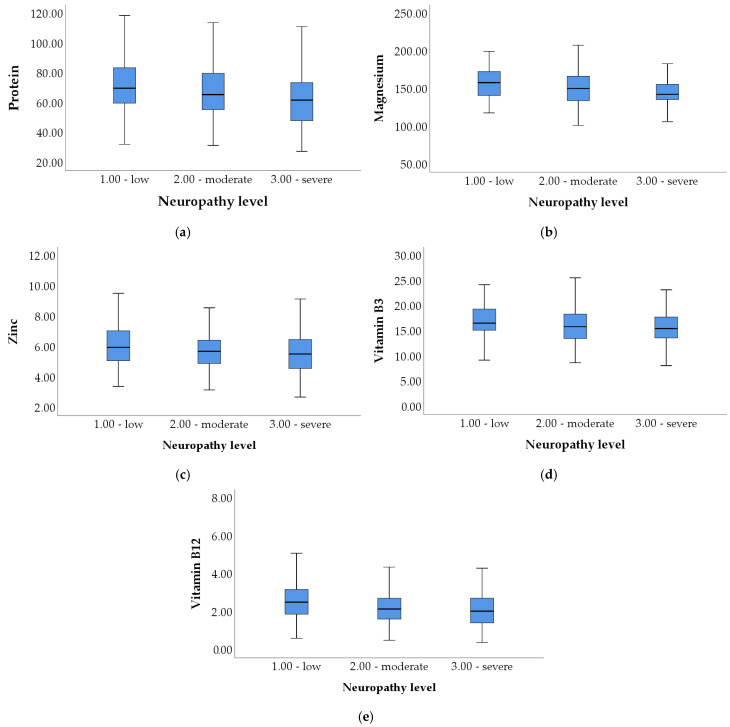
Distribution of nutrient intake according to neuropathy severity. Panels show the nutrients for which significant differences were observed between participants with low, moderate, and severe neuropathy (*p* < 0.05): (**a**) protein, (**b**) magnesium, (**c**) zinc, (**d**) vitamin B3, and (**e**) vitamin B12. In each boxplot, the central line represents the median, and the box indicates the interquartile range (IQR).

For further analysis, only significant variables (*p* < 0.05) were considered, according to the Jonckheere–Terpstra test. Protein, magnesium, zinc, vitamin B3, and vitamin B12 intakes showed a decreasing trend across neuropathy categories, with lower median values observed in patients with more severe neuropathy (Figure 2).

In unadjusted ordinal logistic regression models (Table 5), higher intake of all investigated nutrients was significantly associated with lower neuropathy severity (Table 5). Specifically, protein intake was inversely associated with neuropathy severity (OR = 0.98, 95% CI: 0.97–0.99, *p* = 0.001), as were magnesium (OR = 0.99, 95% CI: 0.98–1.00, *p* = 0.013), zinc (OR = 0.80, 95% CI: 0.69–0.94, *p* = 0.006), vitamin B3 (OR = 0.94, 95% CI: 0.89–0.99, *p* = 0.022), and vitamin B12 (OR = 0.76, 95% CI: 0.62–0.93, *p* = 0.008). Importantly, these associations remained statistically significant after full adjustment for age, sex, treatment, diabetes duration, and HbA1c. In fully adjusted models, protein (OR = 0.98, 95% CI: 0.96–0.99, *p* = 0.001), magnesium (OR = 0.99, 95% CI: 0.98–1.00, *p* = 0.013), zinc (OR = 0.79, 95% CI: 0.66–0.95, *p* = 0.011), vitamin B3 (OR = 0.93, 95% CI: 0.87–0.99, *p* = 0.021), and vitamin B12 (OR = 0.78, 95% CI: 0.61–0.98, *p* = 0.034) remained independently associated with reduced odds of more severe neuropathy. These findings indicate that higher intake of these nutrients is consistently linked to a lower likelihood of advanced neuropathy, independent of glycemic control and disease duration, suggesting a potential direct role in neuropathy pathophysiology. From a clinical perspective, this is particularly relevant, as current management of diabetic neuropathy primarily focuses on glycemic control, while nutritional factors are often underemphasized. For instance, magnesium and zinc are involved in glucose metabolism and anti-inflammatory pathways, while B vitamins play a key role in nerve conduction and repair. Therefore, optimizing dietary intake of these nutrients may represent a complementary strategy in the prevention or progression of diabetic neuropathy. These results highlight the potential value of targeted nutritional interventions alongside standard metabolic management in patients with diabetes.

From a clinical perspective, these data support a comprehensive approach to neuropathy prevention and management in type 2 diabetes, in which optimization of glycemic control remains the priority, while dietary quality and adequate micronutrient intake may represent complementary targets.

## 4. Discussion

The present study shows that several nutrients, including protein, magnesium, zinc, vitamin B3, and vitamin B12, were independently associated with lower neuropathy severity, even after adjustment for glycemic control and diabetes duration. These findings suggest that specific components of the diet may influence neuropathy severity beyond traditional metabolic factors [19]. In contrast, the attenuation of associations observed for dietary patterns after adjustment indicates that their effects may be largely mediated through glycemic exposure and disease progression rather than direct biological mechanisms. Clinically, these findings suggest that dietary patterns are not independently associated with neuropathy severity but rather reflect underlying metabolic control. Glycemic status and diabetes duration appear to be the primary drivers of neuropathy progression, while diet may influence neuropathy indirectly through its impact on metabolic regulation [19].

The results indicated that unfavorable dietary patterns, characterized by high consumption of processed foods, refined carbohydrates, and alcohol, are associated with greater neuropathy severity, whereas healthier dietary patterns appear to be inversely associated. This supports the hypothesis that diet-related metabolic and inflammatory mechanisms may contribute to the development and progression of neuropathy [10]. Additionally, the observed associations may reflect bidirectional relationships, as individuals with more severe neuropathy may adopt less healthy eating behaviors due to functional limitations and reduced quality of life [20]. These associations may be explained by several interconnected biological mechanisms. Dietary patterns such as the Mediterranean diet have been associated with reduced oxidative stress and chronic low-grade inflammation through their high content of antioxidants, polyphenols, dietary fiber, and micronutrients [7]. These effects may improve insulin sensitivity, support glycemic regulation, and reduce the metabolic disturbances that contribute to peripheral nerve injury [7,13]. In contrast, Western-type dietary patterns have been linked to increased insulin resistance, oxidative stress, and activation of pro-inflammatory pathways, all of which may exacerbate neuronal dysfunction and accelerate diabetic neuropathy progression [9]. Persistent hyperglycemia can further promote the formation of advanced glycation end-products, mitochondrial dysfunction, and microvascular damage, leading to impaired nerve perfusion, demyelination, and axon degeneration [9]. Therefore, the observed associations between dietary patterns and neuropathy severity may reflect not only differences in glycemic control but also broader effects on inflammatory, oxidative, and neurodegenerative processes involved in diabetic nerve damage. From a practical perspective, these mechanisms may be linked to specific food choices. Higher consumption of vegetables, fruits, legumes, whole grains, nuts, and olive oil (Mediterranean diet) may provide antioxidants, polyphenols, dietary fiber, magnesium, and B vitamins that support metabolic health and neuronal function [7]. Conversely, frequent consumption of refined grains, sugar-sweetened products, processed foods, red and processed meats, and foods rich in saturated fats (Western diet) may promote systemic inflammation, oxidative stress, and insulin resistance, thereby contributing to the progression of diabetic complications, including neuropathy [9]. These observations support the concept that dietary quality, beyond individual nutrient intake, may play an important role in modulating pathways involved in diabetic nerve damage. The results regarding fat consumption indicated that although shifts in fat sources may be observed descriptively, they do not appear to play a significant independent role in neuropathy severity in this population.

The independent associations observed for micronutrients are supported by the literature. Magnesium plays a key role in neuronal excitability, membrane stability, and glucose metabolism, and its deficiency has been linked to impaired nerve function and increased risk of diabetic neuropathy [21]. Zinc exerts antioxidant and anti-inflammatory effects and is involved in cellular repair processes, which are critical in the context of chronic nerve damage [22]. Similarly, B vitamins are essential for neuronal metabolism, myelin synthesis, and neurotransmission, and their deficiency is a well-established cause of neuropathy [23]. Evidence from clinical and experimental studies suggests that these micronutrients may contribute to improved nerve conduction and reduced neuropathic symptoms, even in the absence of significant changes in glycemic control [24].

Although some of the observed odds ratios were numerically close to unity, particularly for protein and magnesium intake (Table 5), these estimates should be interpreted in the context of the measurement scale used in the analyses. The reported odds ratios represent the change in neuropathy severity associated with a one-unit increase in nutrient intake, whereas real-world dietary modifications typically involve substantially larger changes in consumption [7,25]. Consequently, even modest per-unit effects may translate into more meaningful differences when accumulated over time or across broader dietary changes. Furthermore, diabetic neuropathy is a multifactorial complication influenced by numerous metabolic, vascular, and inflammatory pathways; therefore, individual nutrients would not be expected to exert large independent effects [7,26]. From a clinical perspective, improving overall dietary quality and ensuring adequate intake of key micronutrients may contribute to a comprehensive strategy for reducing neuropathy risk and progression.

Previous research on diet and diabetic neuropathy has predominantly focused on predefined dietary indices or isolated nutrient deficiencies, with relatively limited attention to neuropathy severity as a graded outcome. For example, studies have shown associations between individual micronutrients and nerve function, particularly vitamin B12, whose deficiency is well established as a cause of peripheral neuropathy [24]. Similarly, magnesium deficiency has been linked to impaired nerve conduction and increased risk of diabetic complications through its role in glucose metabolism and neuronal stability [27]. Zinc has also been implicated in oxidative stress modulation and neuronal repair processes, which are critical in the pathogenesis of diabetic neuropathy [22]. However, most of these studies have not simultaneously evaluated overall dietary patterns and nutrient-level exposures within the same analytical framework. Our findings extend the current literature by demonstrating that, although dietary patterns may reflect broader lifestyle behaviors, their possible associations with neuropathy severity appear to be largely mediated by glycemic factors, whereas specific micronutrients remain independently associated. This dual-level analysis provides a more nuanced understanding of diet–neuropathy relationships and supports the hypothesis that targeted nutritional components may have direct effects on neural integrity beyond global dietary behaviors. Recent evidence underscores the importance of dietary factors in neuropathy progression, largely driven by their effects on metabolic control [19]. Our findings extend this perspective by demonstrating that specific micronutrients remain associated with neuropathy severity independently of glycemic factors. Early-life nutritional exposures may play a role in shaping long-term metabolic risk, as suggested by recent evidence showing that infant feeding type is associated with the development of diabetes [28], where formula feeding was more frequent among affected individuals. In this context, our findings extend the role of nutrition across the life course, demonstrating that not only early but also adult dietary exposures, particularly micronutrient intake, could be associated with the severity of diabetic complications.

The study also has some limitations. These results should be interpreted in light of the cross-sectional design, which hinders causal inference, and the use of FFQ-based dietary assessment, which may introduce measurement error. However, the consistency of associations across adjusted models and the biological plausibility of the observed relationships strengthen the relevance of our findings. In addition, several potentially relevant confounding factors, including body mass index, smoking status, alcohol consumption, physical activity, lipid profile, and socioeconomic status, were not included in the final models. Consequently, residual confounding cannot be excluded, and the observed associations should be interpreted with caution. Future longitudinal and interventional studies are needed to clarify whether targeted nutritional strategies may contribute to the prevention or progression of diabetic neuropathy.

## 5. Conclusions

The present study suggests that, in patients with type 2 diabetes, the relationship between overall dietary patterns and diabetic neuropathy severity is largely explained by major clinical and metabolic factors, particularly diabetes duration and glycemic control. By contrast, higher intake of protein, magnesium, zinc, vitamin B3, and vitamin B12 remained independently associated with lower odds of more severe neuropathy. These findings support the view that, although dietary patterns may reflect the general metabolic milieu, specific nutritional factors may be more closely linked to neuropathy severity. Further prospective studies are needed to determine whether these associations are causal and whether targeted nutritional strategies may improve neuropathy outcomes. These findings highlight the importance of moving beyond global dietary patterns and considering specific nutrient exposures when evaluating diet–neuropathy relationships.

## Figures and Tables

**Table 1:** Characteristics of the population studied.

Characteristic	Neuropathy Level (n = 300)			*p*
	1.00	2.00	3.00	
Neuropathy level	83 (27.7%)	132 (44.0%)	85 (28.3%)	-
Age (years)	66 (41–85)	67 (45–85)	67 (22–90)	0.28 ^a^
Diabetes duration (years)	3 (0.1–30)	10.50 (1–32)	17 (0.3–40)	<0.001 ^a^
HbA1c (%)	6.50 (4.6–9.0)	7.10 (5.3–10.8)	8.70 (7.2–15.8)	<0.001 ^a^
***Sex***				
Men	38 (45.8%)	65 (49.2%)	37 (43.5%)	0.70 ^b^
Women	45 (54.2%)	67 (50.8%)	48 (56.5%)	0.70 ^b^
***Treatment***				
Oral antidiabetic drugs	76 (91.6%)	100 (75.8%)	28 (32.9%)	<0.001 ^b^
Insulin	1 (1.2%)	8 (6.1%)	26 (30.6%)	<0.001 ^b^
Combined	6 (7.2%)	24 (18.2%)	31 (36.5%)	<0.001 ^b^

^a^ according to the Mann–Whitney test; ^b^ according to the χ^2^ test.

**Table 2:** Jonckheere–Terpstra test results for food patterns.

Pattern	Number of Levels in Neuropathy	N	Observed J–T Statistic	Mean J–T Statistic	Std. Deviation of J–T Statistic	Std. J–T Statistic	*p* (2-Tailed)
Western/Fast food	3	300	16,424.00	14,615.50	809.02	2.24	0.03
Healthy/prudent	3	300	12,141.50	14,615.50	809.09	−3.06	0.01
Refined grains and starches	3	300	16,226.50	14,615.50	808.22	1.99	0.04
Light meal and sweet spreads	3	300	14,372.00	14,615.50	809.04	−0.30	0.76
Alcohol and animal fat	3	300	16,169.50	14,615.50	808.06	1.92	0.05

**Table 3:** Ordinal regression models for food patterns.

Dietary Pattern	Model	B (SE)	OR (95% CI)	*p*-Value
Western/fast-food	Unadjusted	0.234 (0.109)	1.26 (1.02–1.56)	0.03
	Adjusted	−0.032 (0.165)	0.97 (0.70–1.34)	0.85
Healthy/prudent	Unadjusted	−0.352 (0.110)	0.70 (0.57–0.87)	0.01
	Adjusted	−0.129 (0.159)	0.88 (0.64–1.20)	0.42
Refined grains and starches	Unadjusted	0.211 (0.108)	1.24 (1.00–1.53)	0.05
	Adjusted	0.038 (0.150)	1.04 (0.77–1.39)	0.80
Alcohol and animal fat	Unadjusted	0.230 (0.109)	1.26 (1.02–1.56)	0.03
	Adjusted	0.208 (0.168)	1.23 (0.89–1.71)	0.22

B = regression coefficient; OR = odds ratio (exp(B)); CI = confidence interval. Adjusted models include age, sex, treatment, diabetes duration, and HbA1c. Statistical significance was set at *p* < 0.05.

**Table 4:** Jonckheere–Terpstra test results for nutrient intake.

Nutrient	Number of Levels in Neuropathy	N	Observed J–T Statistic	Mean J–T Statistic	Std. Deviation of J–T Statistic	Std. J–T Statistic	*p* (2-Tailed)
Protein	3	300	12,024.000	14,615.500	809.695	−3.201	<0.01
Lipids	3	300	14,351.000	14,615.500	809.695	−0.327	0.74
Fiber	3	300	15,090.000	14,615.500	809.695	0.586	0.56
Carbohydrates	3	300	16,165.000	14,615.500	809.695	1.914	0.06
Ca	3	300	13,539.000	14,615.500	809.695	−1.330	0.18
Mg	3	300	12,200.000	14,615.500	809.695	−2.983	0.01
Fe	3	300	13,607.000	14,615.500	809.695	−1.246	0.21
Zn	3	300	12,588.000	14,615.500	809.695	−2.504	<0.01
Na	3	300	14,480.000	14,615.500	809.695	−0.167	0.87
K	3	300	13,521.000	14,615.500	809.695	−1.352	0.18
Vit A	3	300	15,801.000	14,615.500	809.695	1.464	0.14
Vit C	3	300	15,712.000	14,615.500	809.695	1.354	0.18
Vit D	3	300	13,353.000	14,615.500	809.554	−1.560	0.12
Vit E	3	300	14,259.000	14,615.500	809.695	−0.440	0.66
Vit B1	3	300	13,705.000	14,615.500	809.695	−1.124	0.26
Vit B2	3	300	13,181.000	14,615.500	809.695	−1.772	0.08
Vit B3	3	300	12,634.000	14,615.500	809.695	−2.447	0.01
Vit B6	3	300	13,132.000	14,615.500	809.695	−1.832	0.07
Folate	3	300	14,128.000	14,615.500	809.695	−0.602	0.55
Vit B12	3	300	12,357.000	14,615.500	809.695	−2.789	<0.01

**Table 5:** Ordinal regression model for nutrient intake.

Nutrient	Model	B (SE)	OR (95% CI)	*p*-Value
Protein	Unadjusted	−0.019 (0.006)	0.98 (0.97–0.99)	<0.01
	Adjusted	−0.022 (0.007)	0.98 (0.96–0.99)	0.01
Magnesium	Unadjusted	−0.011 (0.004)	0.99 (0.98–1.00)	0.01
	Adjusted	−0.012 (0.005)	0.99 (0.98–1.00)	0.01
Zinc	Unadjusted	−0.218 (0.079)	0.80 (0.69–0.94)	0.01
	Adjusted	−0.234 (0.092)	0.79 (0.66–0.95)	0.01
Vitamin B3	Unadjusted	−0.064 (0.028)	0.94 (0.89–0.99)	0.02
	Adjusted	−0.074 (0.032)	0.93 (0.87–0.99)	0.02
Vitamin B12	Unadjusted	−0.276 (0.105)	0.76 (0.62–0.93)	0.01
	Adjusted	−0.254 (0.120)	0.78 (0.61–0.98)	0.03

B = regression coefficient; OR = odds ratio (exp(B)); CI = confidence interval. Adjusted models include age, sex, treatment, diabetes duration, and HbA1c. Statistical significance was set at *p* < 0.05.

## Data Availability

The raw data supporting the conclusions of this article will be made available by the authors on request.

## Supplementary Materials

The following supporting information can be downloaded at https://www.mdpi.com/article/10.3390/nu18132134/s1, Figure S1: FFQ questionnaire; Table S1: PCA results.
